# Effect of carbon nanotubes loading and pressure on the performance of a polyethersulfone (PES)/carbon nanotubes (CNT) membrane

**DOI:** 10.1038/s41598-021-03042-z

**Published:** 2021-12-10

**Authors:** Mlungisi Martin Ngoma, Machodi Mathaba, Kapil Moothi

**Affiliations:** Department of Chemical Engineering, Faculty of Engineering and the Built Environment, University of Johannesburg, 37 Nind Street, Doornfontein, Johannesburg, 2028 South Africa

**Keywords:** Carbon nanotubes and fullerenes, Nanoparticles, Environmental, health and safety issues, Chemical engineering, Polymer chemistry

## Abstract

This paper focuses on modifying a PES membrane with acid-functionalised carbon nanotubes (CNT) for industrial wastewater treatment. Embedding acid functionalised carbon nanotubes (CNTs) within the membrane matrix would increase the membrane flux by increasing the membrane pore size and surface area, rejection and thermal stability. Pure PES membranes were prepared by phase inversion method and infused with CNTs at 2.5, 5, 7.5 and 10 wt% loading to fabricate PES/2.5 wt% CNT, PES/5 wt% CNT, PES/7.5 wt% CNT and PES/10 wt% CNT membranes respectively. Characterisation was performed using Transmission Electron Microscopy (TEM) to determine CNT morphology, X-ray Diffraction (XRD) to determine the functional groups attached to CNTs, Thermogravimetric Analysis (TGA) to determine the thermal stability of the membranes, Scanning Electron Microscope (SEM) to determine membrane morphology, Bunauer-Emmett-Teller (BET) method to obtain pore size information and Contact Angle (CA) to determine the membrane hydrophilicity. Membrane performance was then evaluated with a dead-end stirred cell using industrial wastewater containing traces of Cu, Fe, Ni, Zn and Cl. Permeate flux results showed a direct proportion relationship with increasing CNT loading and increasing pressure (100 kPa, 300 kPa, 500 kPa, 700 kPa, 900 kPa and 1100 kPa). PES/5 wt% CNT membrane showed the most enhanced performance compared to the other membranes, achieving reasonably high flux of 43.7 L/m^2^h and rejection of 89.6% Cu, 100% Fe, 90.5% Ni, 68.8% Zn and 99.99% Cl at 300 kPa. The results obtained showed that the PES membrane embedded with functionalised CNTs could be used for the treatment of industrial wastewater.

## Introduction

The preservation of fresh water resources for domestic, agricultural and industrial use is a crucial part of human, animal and environmental life. However, discharging industrial effluent or wastewater that contains heavy metals and salts into open water surfaces such as rivers, sewers and reservoirs without treatment raises both health and scientific concerns. Large scale industries such as metal plating, metallurgy, mining, chemicals manufacturing, steel, battery and plastics manufacturing are the main contributors of discharging heavy metals and salts in open surfaces^1^. The aforementioned industries produce heavy metals such as nickel, copper, zinc, lead, mercury, gold and chromium (IV)^1,2^. When present in water, these contaminants entail severe health complications to the consumer such as organ damage and cancer^2^.

Traditional wastewater treatment methods such as flocculation, sedimentation, coagulation, screening and aerobic and anaerobic treatment are ineffective in the removal of salts and trace metals^3,4^. Membrane technology has made a significant breakthrough compared to the aforementioned methods for the reason that it is operationally cost effective, less energy consuming, has the ability to be design-flexible and has high removal capacity^5,6^. Pressure driven membrane technologies currently in use are based on permeability, selectivity and membrane pore size and can be classified as follows: microfiltration (rejects solid particles like colloids, macromolecules and bacteria in the size range of 1 µm-0.1 µm) ultrafiltration (rejects macromolecules and high molecular weight solutes in the particle size range of 0.1 µm–0.01 µm), nano-filtration (rejects divalent and monovalent ions in the size range of 0.01 µm–0.001 µm) and Reverse Osmosis (RO)^3,4^.

Polymeric composite membranes have been reported to be effective in the removal of heavy metals and salts from industrial wastewater (containing copper (Cu), iron (Fe) and nickel (Ni), particularly in Ultrafiltration (UF) and Nanofiltration (NF) processes due to having have high selectivity, permeability, thermal and chemical stability and good film forming properties, and can be modified to fit MF, UF and NF requirements^5–11^. Of the various polymers available, Polysulfone (PSF) and Polyethersulfone (PES) emerge as leading polymer choices for membrane filtration partly due to their high thermal and chemical stability, and the permeability of membranes fabricated from PES and PSF^12,13^. Polyethersulfone (PES) membranes have high thermal and chemical stability, however, due to its relatively high hydrophobic nature (owing to sulfonyl functional group in the PES structure), pure PES membranes are therefore prone to fouling and lacking in UF and NF applications due to high permeability, and inability to remove monovalent and divalent ions effectively^12,13^. PES has a higher degree of hydrophilicity than PSF and is therefore a more desirable polymer for the application of a polymer composite membrane for the treatment of industrial effluent^7,11–13^.

The limiting factor of polymeric membranes is that they are generally hydrophobic, thus making polymeric membranes very susceptible to fouling^5,11^. Membrane Fouling can be described as the accumulation and adsorption or dissolution of colloids, particles, salts, divalent and monovalent ions on the surface and in the pores of the membrane^4,7,11^. The foulants contained in industrial effluent are of colloidal, biological, organic and inorganic nature, by polarisation they are then attracted to the hydrophobic membrane surface and pores^5^. Fouling leads to a drastic decline in the lifespan of the membrane by reducing the permeation and selectivity capacities of the membrane^4,7,11^. The most common types of fouling among polymeric membranes such as PES are colloidal fouling, scaling, inorganic, organic and biofouling^7,11^.

In order to mitigate and reduce fouling while improving flux and rejection, researchers have sought to improve the membrane hydrophilicity and morphology by blending functionalised CNTs into the polymer matrix. The acid functionalisation of CNTs has been reported by Celik et al.^14^ to improve the rejection and flux of the composite due to the interaction of the carboxylic (-COOH) functional groups with wastewater constituents. This interaction was explained by Son et al.^15^ and Wiley^16^ and to be associated with the surface charge density and the obtaining of an overall negative charge on the membrane surface therefore leading the membrane to repel like-charged ions and attract opposite-charged ions. Das et al.^10^ and Wang^17^ found and reported that the addition of –COOH and –OH groups on the ends of the CNTs enhance the adsorption of polar compounds and decrease in the adsorption of organic matter on the surface of the membrane by activating hydrophilic sites for water cluster formation. Furthermore, the addition of acid functionalised CNTs is critical to the rejection ability of polymeric composite membranes by giving rise to strong repulsion forces that aid the sieving ability of the membrane whereby molecular sizes increase in solution i.e. sizes increase from left to right in the periodic table, however in solution cations become larger^15,18,19^. Generally, membrane surface gains a negative charge when the membrane comes into contact with alkaline wastewater, this enables the membrane to repel anions and attract cations to adsorb on the membrane surface, therefore a membrane infused with acid functionalised CNTs employs adsorption and size selectivity^15,18,19^.

Maphutha et al.^4^ observed an improvement in flux, rejection and anti-fouling properties after blending CNTs into PSF. Similarly, Rameetsi et al.^20^ observed increased rejection and flux after functionalised CNT into a 75:25 PES/PSF polymer. Wang et al.^21^ observed an increase in permeate flux and also salt rejections of 87%, 72% and 24% for Na_2_SO_4_, MgSO_4_, and NaCl respectively after blending acid-functionalised CNTs into PES. Moreover, Nikita et al.^22^ also reported metal (Cu^2+^) rejection of 78% for a PES/CNT polymer.

This research investigated the effect of CNT loading on the performance of a PES/CNT membrane by evaluating the change in physiochemical properties, thermal stability, rejection and flux of the composite at various CNT loadings.

## Materials and methods

### Materials

Pristine multi-walled carbon nanotubes (MWCNTs, 95–98% carbon basis, 50–90 nm outer diameter, Length 5–9 μm) were purchase from Merck (Sigma-Aldrich) South Africa, polyethersulfone granules (PES; 3 mm nominal granule size; 58,000 g/mol), and dimethyl sulfoxide (DMSO; > 99.7% for HPLC) solvent were also purchased from Merck (Sigma-Aldrich) South Africa. Analytical grade 55% nitric acid (HNO_3_) and 98% sulfuric acid (H_2_SO_4_) were purchased from ACE Chemical Enterprises (South Africa).

### Pure PES membrane fabrication

A 10 wt% pure PES membrane was prepared by dissolving PES granules in Dimethyl Sulfoxide (DMSO). The mixture was allowed to stir on a magnetic equipped stirrer for 24 h until homogeneity was achieved. Thereafter, the gel was cast on a flat glass plate using a casting knife. The glass plate containing the casting gel was placed in DI water for 2 min to form a flat sheet PES membrane by phase inversion method. The cast membrane was allowed to rest in water for 12 h to allow for the removal of the solvent and thereafter the membrane was dried in ambient air for 24 h^11,14,17,23,24^.

### Functionalisation of CNT

A mixture 200 mL of 95% Sulphuric acid (H_2_SO_4_) and 55% Nitric acid (HNO_3_) was prepared following a ratio of 3:1. Thereafter, pristine CNTs were added to the acid mixture, which was sonicated in an ultrasonicator for 3 h at 70 °C to improve the dispersion of the CNTs and to attach hydroxyl (-OH) and carboxyl (-COOH) groups on the ends of the CNTs. The CNTs were then filtered from the acid mixture, washed with DI water until the pH of the water reached 7. The CNTs were then dried at 500 °C for 12 h in an oven^5,7,11,14,17,23–25^.

### Formation of PES/CNT composite

PES granules were dissolved in DMSO to make up 10 wt% PES casting gels. After the granules were dissolved; 2.5wt%, 5wt%, 7.5wt% and 10 wt% of –COOH functionalised CNTs were added into respective polymer casting gel mixtures. The respective casting gel mixtures were allowed to stir on a magnetic equipped stirrer for 24 h until homogeneity was achieved in order to fabricate PES/2.5 wt% CNT, PES/5 wt% CNT, PES/7.5 wt% CNT and PES/10 wt% CNT casting gels. The gels were respectively cast on flat glass plates using a casting knife. The glass plates containing the respective casting gels were then placed in DI water for 2 min to form flat sheet PES/2.5 wt% CNT, PES/5 wt% CNT, PES/7.5 wt% CNT and PES/10 wt% CNT membranes by phase inversion method. The cast membranes were allowed to rest in DI water for 12 h to ensure removal of the solvent and thereafter the membrane was dried in ambient air for 24 h^11,14,17,23–25^.

### Characterisation

The CNTs were characterised using the Transmission Electron Microscopy (TEM), particularly the FEI TECNAI G^2^ Spirit electron microscope to study the morphology and identity of the CNT. X-ray Diffraction (XRD) was used to confirm the success of the acid functionalisation by identifying the functional groups present on the pristine and functionalised CNTs. The pure PES, PES/2.5 wt% CNT, PES/5 wt% CNT, PES/7.5 wt% CNT and PES/10 wt% CNT membranes were characterised using the Scanning Electron microscopy (SEM) model JEOL JSM-IT100-SEM to determine the change in morphology caused by the addition of CNTs, while the Bunauer-Emmett-Teller (BET) method and Secondary Electron Detector (SED) contained in the SEM were used to obtain pore size information of the membranes using the same SEM model. Contact Angle analysis was done by the sessile drop method to investigate the wettability of the PES, PES/2.5 wt% CNT, PES/5 wt% CNT, PES/7.5 wt% CNT and PES/10 wt% CNT membranes using the Data-physics OCA-Series instrument to obtain the results. Thermogravimetric analysis (TGA) for the determination of the thermal stability of the PES, PES/2.5 wt% CNT, PES/5 wt% CNT, PES/7.5 wt% CNT and PES/10 wt% CNT membranes was done using the TA Instruments Universal Analysis 2000 under air flowrate of 25 mL/min, temperature rate of 30 °C/min and a temperature range of 0 °C—900 °C for a sample of 3.5 mg.

### Filtration experiments

The filtration experiments were conducted using the Sterlitech HP4750 dead-end stirred cell with an effective filtration surface area of 0.00126 m^2^ and a cell capacity of 800 mL. As such, membranes with a radius of 0.02003 m were prepared. The membranes were each compacted with DI water at 1100 kPa prior to individual testing. After compacting, 750 mL of industrial effluent containing 30 ppm Cu, 7 ppm Fe, 0.64 ppm Ni, 0.08 ppm Zn and 1976.4 ppm Cl was added into the stirred cell. The industrial wastewater was collected from the municipal discharge point at an electroplating and surface finishing effluent treatment plant in Centurion, South Africa. Each of the membranes (PES/2.5 wt% CNT, PES/5 wt% CNT, PES/7.5 wt% CNT and PES/10 wt% CNT) were tested at transmembrane pressures of 100 kPa, 300 kPa, 500 kPa, 700 kPa, 900 kPa and 1100 kPa using the industrial wastewater. The filtration apparatus consisted of Nitrogen gas (N2) which was used to provide a driving force within the filtration cell, the filtration cell, a magnetic stirrer to improve solid suspension and homogeneity. The filtrate was then analysed using the Agilent technologies 240 Series AA–Model 240 AA Atomic absorption spectroscopy (AA-S). Equation 1 and 2 below were respectively used to determine the membrane flux and rejection for each individual constituent contained in the industrial wastewater^15,20,26^.

$$J=\frac{V}{A\Delta t}$$

$$\mathrm{\%}R=\left(1-\frac{C1}{C0}\right)*100$$

where J is the flux which is the wastewater flow through the membrane over an area overtime (L/m^2^h), V is the volume of filtrate over time (L), A is the surface area of the membrane, ∆t is the time taken to accumulate a volume of filtrate (h), R is the amount of salt or metal ions rejected by the membrane (%), C_0_ is the initial concentration of the salt or metal ion constituent in the wastewater (ppm) and C_1_ is the final concentration of that particular ion (ppm).

## Results and discussions

### Physiochemical characterisation

#### TEM analysis of pristine CNTs

**Figure 1:**
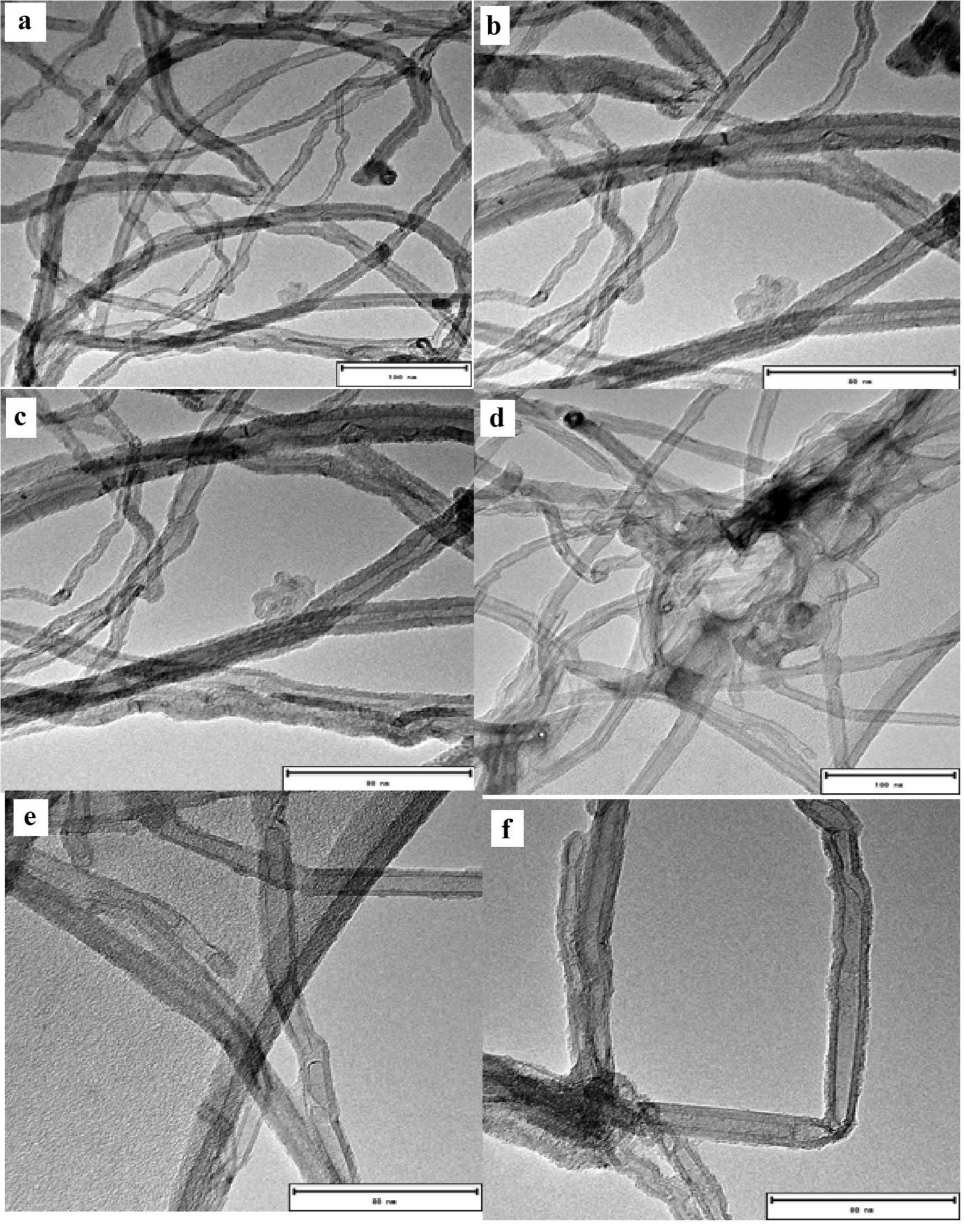
TEM micro images (**a**) Pristine CNTs at × 100 nm magnification (**b**) Pristine CNTs at × 80 nm magnification (**c**) Pristine CNTs at × 80 nm magnification (**d**) Functionalised CNTs at × 100 nm magnification (**e**) Functionalised CNTs at × 80 nm magnification (**f**) Functionalised CNTs at × 80 nm magnification.

From TEM images in Fig. 1, the nature of the nanoparticles was confirmed to be CNTs, by that the CNTs are shown to be hollow, coxial and conical in orientation, entangled (indicating appropriate density and possible agglomeration), multi-walled and were found to have an outer diameter of 80 nm and inner diameter of 12 nm which is synonymous with what was found in other studies^25^. Furthermore, the dark spots visible on the pristine CNTs as indicated in Fig. 1(a–c) can be attributed to the low presence of deactivated catalyst and low amorphous carbon^25^. The functionalisation of CNTs with acids, particularly H_2_SO_4_-HNO_3_, has been reported to remove metallic and catalytic matter from inner walls of CNTs, resulting in open inner cavities within the walls and opening the closed-ends of CNT^5,25,27^. Images of functionalised CNTs are presented in Fig. 1(d–f), and can be seen to be generally without carbon soot and amorphous carbon which was represented by dark spots, thus symbolising successful purification, oxidation and removal of deactivated catalyst. Furthermore, the functionalised CNTs can be observed to have maintained their morphological structure with reduction of surface defects^27^. According to Mazov et al.^27^, acid functionalised CNTs that are of a coaxial conical orientation accelerate efficient adsorption of metal ions on CNT sites.

3.1.2 XRD Analysis of CNTs.

XRD analysis was conducted in order to determine the effectiveness of the functionalisation with the intention to deposit carboxylic (COOH) functional groups onto the CNT ends with a 3:1 mixture of H_2_SO_4_-HNO_3_. Similar analysis was done for pristine (un-functionalised) CNT for the purposes of comparison.

**Figure 2:**
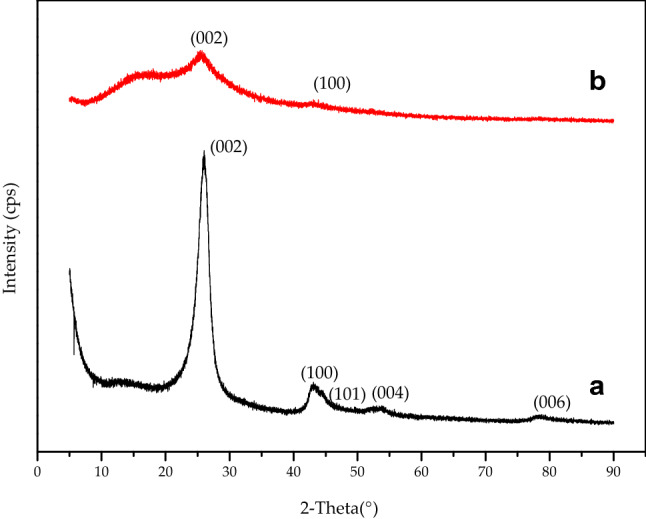
XRD patterns (**a**) Pristine CNT (**b**) Functionalized CNT.

Considering Fig. 2(a), the peak at 2θ = 26.1° corresponds to the (002) reflection which represents stacked graphene sheets, further confirming MWCNT. Figure 2(b) shows a reduced peak of the (002) reflection at 2θ = 25.9, according to Khani^28^ and Das et al.^29^, the reduction at this reflection between pristine (un-functionalised) and functionalised CNT peaks represents the removal of carbon related impurities thus implying successful functionalisation. Both un-functionalised and functionalised CNT showed peaks of the (100) reflection at 2θ = 42.2 2θ = 43.4 respectively. The (100) reflection represents hexagonal plane alignment and regularity and this result suggests that the lattice structure in both is parallel and has good crystallinity, further concluded the functionalisation successfully removed amorphous carbon impurities without damaging the structure of the CNT^28–31^. The peaks at 2θ = 52.1, 2θ = 54.2 and 2θ = 77.76 for (101), (004) and (006) reflections represent the various catalytic impurities present on the surface of un-functionalised CNT. It can be seen from Fig. 2(b) that the impurities peaks have disappeared, further alluding to the success of the acid functionalisation^28–31^. This finding is supported by the TEM images of functionalised CNTs in Fig. 1(d–f) which the removal of impurities.

#### Effect of CNT on membrane morphology

**Figure 3:**
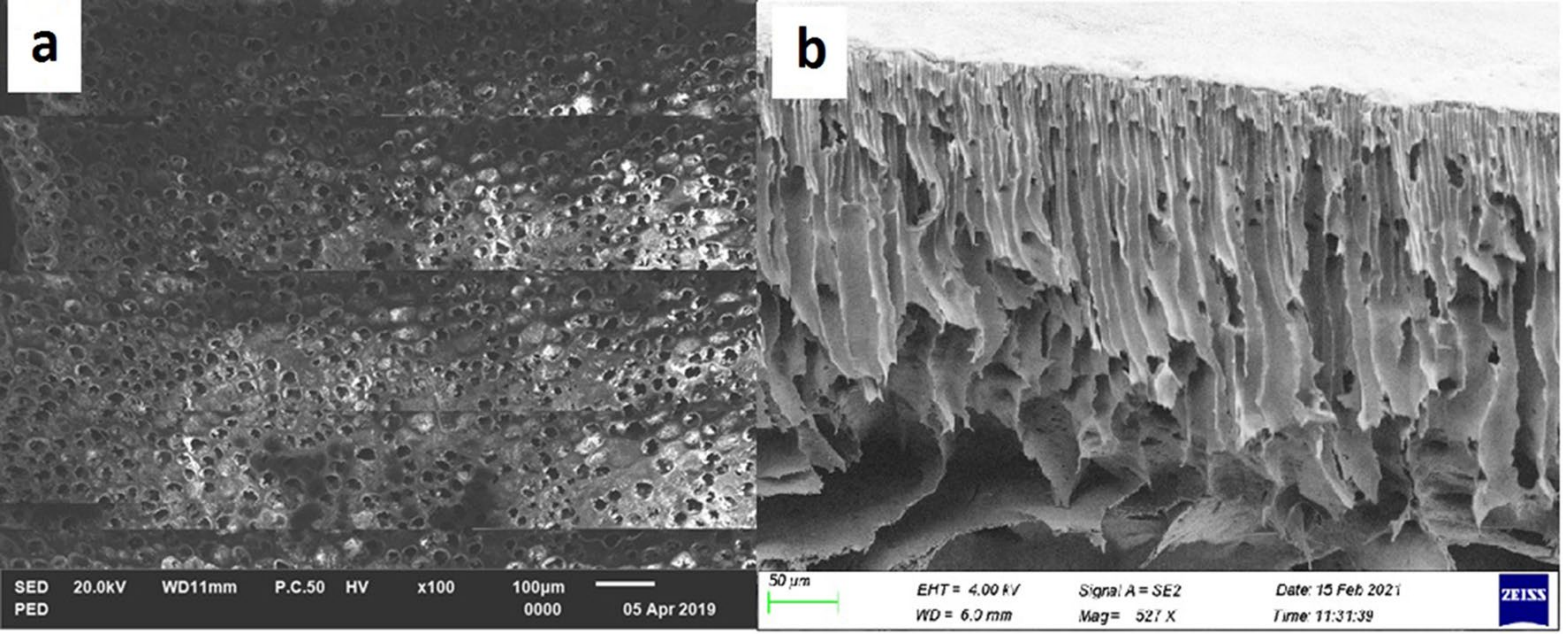
SEM Morphology micro images of pure PES membrane (**a**) surface micro image (**b**) cross sectional micro-image.

As it can be seen from the surface SEM micro image in Fig. 3(a) that the surface of the pure PES membrane is very porous. The pore size of the pure PES membrane was found to be 17.8 nm. Figure 3(b) shows the cross sectional morphology of the pure PES membrane and it can be seen that the membrane has an asymmetric porous membrane structure with a thin dense skin layer with a porous finger like structure. This observation is consistent with findings by Wang et al.^21^ and Vatanpour et al.^19^ who reported that the structure of a pure PES membrane that has been casted by non-solvent induced phase inversion method has a thin dense skin layer and a porous sub layer with macro voids and closed cells in the matrix. The formation of such a structure is due to the instantaneous exchange between the DMSO and DI water in the PES matrix during phase inversion in the water coagulation bath^32^.

**Figure 4:**
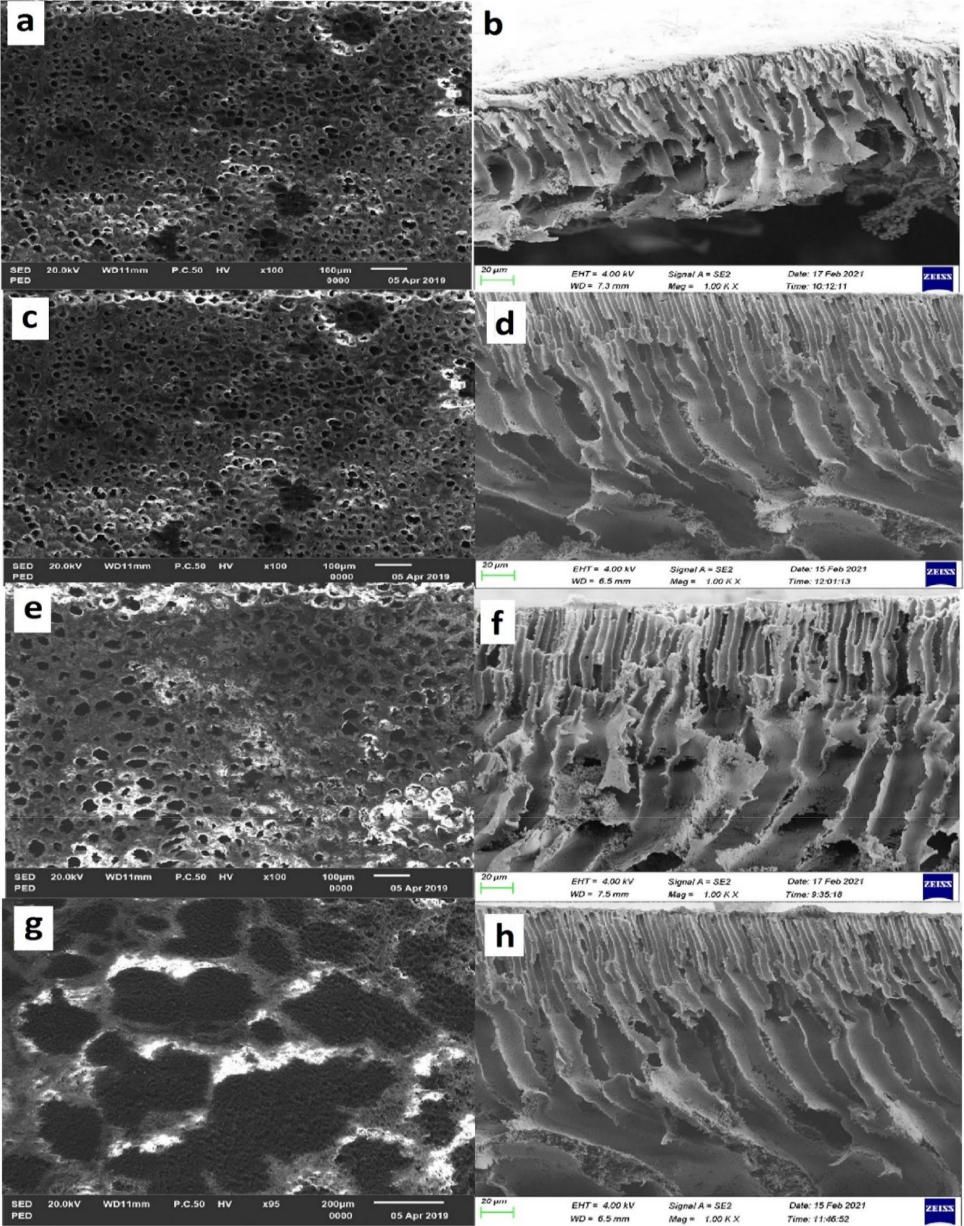
SEM Morphology micro images (**a**) surface SEM for PES/2.5wt% CNT (**b**) cross section SEM for PES/2.5 wt% CNT (**c**) surface SEM for PES/5 wt% CNT (**d**) cross section SEM for PES/5 wt% CNT (**e**) surface SEM for PES/7.5wt% CNT (**f**) cross section SEM for PES/7.5wt% CNT (**g**) surface SEM for PES/10wt% CNT h) cross section SEM for PES/10wt% CNT.

Figure 4(a) and (b) respectively show the surface and cross sectional morphology of PES/2.5 wt% CNT membrane, Fig. 4(c) and (d) respectively show the surface and cross sectional morphology of PES/5 wt% CNT membrane, Fig. 4(a) and (f) respectively show the surface and cross sectional morphology of PES/7.5 wt% CNT membrane; and Fig. 4(g) and (h) respectively show the surface and cross sectional morphology of PES/10 wt% CNT membrane. There is an evident change in the surface and cross sectional morphology with the addition of functionalised CNTs and subsequent increase in CNT loading as seen in Fig. 4 that there is a decrease in the overall number of pores visible on the surface of the PES/CNT membranes and a general increase in the surface pore sizes, with the pores seeming to become more finely dispersed with an increase in CNT loading. BET-SED analysis indicates that relative to the pure PES membrane there was an average pore size increase of 59%, 80%, 116% and 64% for the PES/2.5 wt% CNT, PES/5 wt% CNT, PES/7.5 wt% CNT and PES/10 wt% CNT membranes, respectively. The pore sizes increased with the addition of CNTs but decreased at the highest CNT loading such that the sizes were 28.3 nm ± 7.08, 32.0 nm ± 12.89, 38.4 nm ± 5.48 and 29.2 nm ± 7.99 for pure PES, PES/2.5 wt% CNT, PES/5 wt% CNT, PES/7.5 wt% CNT and PES/10 wt% CNT membranes. The reason for this behaviour is that there is accelerated exchange between the solvent (DMSO) and non-solvent (DI water) at lower CNT loading as a result of low viscosity of the casting gel, thermodynamic immiscibility and wettability of the CNTs, resulting in the formation of larger membrane pores and a porous structure^14,19,32,33^. The effective surface area and macro voids were seen to increase with increasing CNT loading, meaning that the space within the membrane pores becomes increasingly occupied with CNT, thus stretching the membrane pores^14,19,32–34^. The cross sectional SEM images in Fig. 4(b), (d), (f) show that the size of the macro voids formed in the PES substrate increased with increasing CNT loading towards the bottom of the membrane, changing both in size and shape in comparison to the pure PES membrane thus confirming the successful embedding of CNTs into the PES matrix. Furthermore, agglomerates of CNTs can be seen to have formed along the stretch of the PES matrix. The decrease in pore size for the PES/10 wt% membrane is caused by the possible increase in casting gel viscosity as a result of the high loading of 10 wt% causing CNT agglomeration, leading a delay in diffusion during phase inversion (yielding a dense skin layer)^4,33,34^. Similar to this study, Celik et al.^14^; Manawi et al.^33^; Phao et al.^32^, and Vatanpour et al.^19^, fabricated flat sheet blended PES/CNT membranes using phase inversion method by blending –COOH functionalised CNTs into PES and reported findings that are synonymous with this study. These findings are also supported by the filtration results presented in Fig. 7.

#### Effect of CNT loading on membrane thermal stability

**Figure 5:**
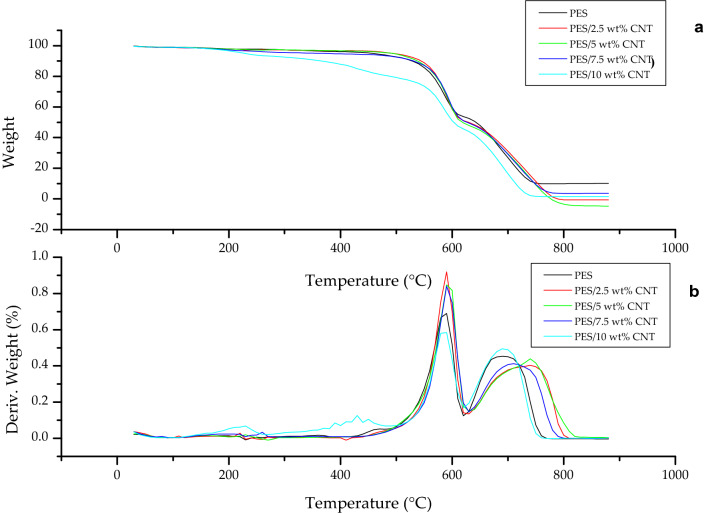
Thermal stability of PES and PES/CNT composites (**a**) Weight loss (**b**) DTA-derivative weight loss.

CNT have also been vastly reported in other studies to improve the thermal stability of CNT composite membranes^35–37^. TGA was conducted to determine the thermal stability of the PES, PES/2.5 wt% CNT, PES/5 wt% CNT, PES/7.5 wt% CNT and PES/10 wt% CNT membranes. The thermal stability is shown by means of weight % change and derivative weight in Fig. 5(a) and (b)

Figure 5(a) indicates that all the PES, PES/2.5 wt% CNT, PES/5 wt% CNT, PES/7.5 wt% CNT and PES/10 wt% CNT membranes suffered a minimal mass loss 1.87, 1.93, 1.93, 2.45, and 2.97% respectively between 35 °C and 200 °C, a loss of less than 3% may indicate consistency in the amount of solvent used in the fabrication of each membrane and adequate drying. This weight loss has been attributed to the loss of DMSO and moisture alike as DMSO and water boil at 189 °C and 100 °C respectively^4^.

All the membranes underwent a multi-stage pyrolysis/degradation, and therefore multiple peaks per membrane curve representing different degradation stages can be seen from Fig. 5(b). The weight loss for the PES membrane between 200 and 450 °C is attributed to the formation of a char layer, which is generally formed by PES under thermal degradation and acts as a protective film for the membrane surface, furthermore it impedes the dissemination of volatile matter^35^. The PES membrane started degrading at 460 °C, and the weight loss here can be attributed to the breaking down of C = C, C-H, and C-H from the phenyl groups; and the formation of volatiles such as CO_2_ and benzene^36,38,39^. The weight loss in the second stage at 640 °C can be attributed to the breaking down of the sulfonyl (SO_2_) group which is the core of the polymer, the ether group and the formation and release of more volatiles such as sulfamic acids and phenols^36,38,39^. The last stage is a classified as the formation of further char from 760 °C to 880 °C, and thus melting of the membrane^35^.

The blending of functionalised CNT into the PES matrix stabilises the formation of the protective char by means of π-π interactions^35,40,41^. In the range of char formation (between 200 °C and 450 °C), the weight loss for PES/2.5 wt% CNT, PES/5 wt% CNT, PES/7.5 wt% CNT and PES/10 wt% CNT is attributed to the carboxylic functional groups that were attached on the tips of the CNT during functionalisation and the release of CO_2_^35,36,39^. The degradation temperature for all the PES/CNT composites can be seen to be improved relative to that of the pure PES membrane. The addition of CNTs into PES increases the rigidity of the composite membrane by reducing the polymer chain mobility, inadvertently increasing the glass transition temperature of the composite membrane, thus increasing the membrane thermal stability^25,37,42^. As such, the membrane thermal stability increased linearly when 2.5 wt% CNT, 5 wt% CNT and 7.5 wt% were added to PES. However, a CNT loading of 10 wt% yielded the lowest thermal stability due to CNT agglomeration. Previous studies have reported that higher CNT loadings are prone to accelerating agglomeration within the membrane with increases the polymer free volume, thus decreasing membrane rigidity^25,37,42^. Furthermore, studies also reported a trade-off between rigidifying the membrane due to CNT addition and membrane softening due to porous microstructure, as such higher CNT loadings are prone to yield porous membranes and softer membranes^25,37,42,43^. The order of highest thermal stability was such that PES/7.5 wt% CNT > PES/5 wt% CNT > PES/2.5 wt% CNT > PES > PES/10 wt% CNT. The degradation temperature of the PES/7.5 wt% CNT membrane was increased to 490 °C whereby the breaking down of C = C, C-H, and C-H derived from phenyl groups occurred, while the destruction of the sulfonyl group started at 630 °C^35,36,39^. This increase in thermal stability is synonymous with results in other studies^37,42,43^. Lastly, CNT improves the thermal stability by reducing the polymer chain mobility, thereby making the membrane more rigid, and by obstructing of the flux of degradation^25,43^.

#### Effect of CNT loading on membrane degree of wettability (hydrophilicity)

**Figure 6:**
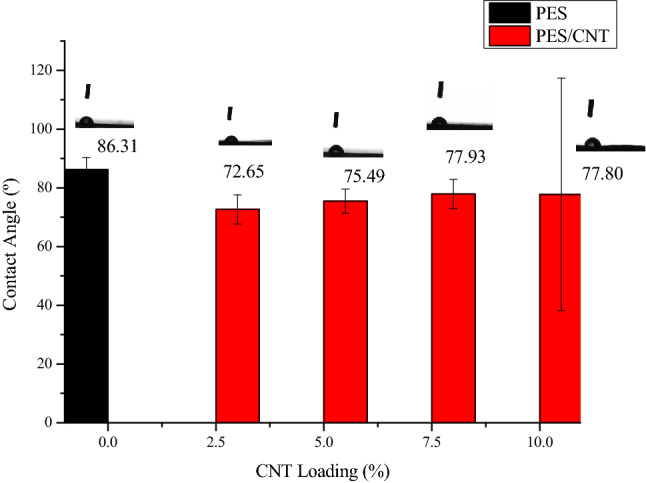
Contact angle measurement.

The hydrophilicity was determined by measuring contact angle, and in general a decrease increase in contact angle indicates an increase in hydrophilicity^21,32^. This is a very important property in the improvement of flux as the membrane’s polarity is increased and thus attracts more wastewater molecules^15^. Furthermore, it has been reported in other studies^15^ that improved hydrophilicity improves in the rejection of non-polar foulants such as suspended colloids and proteins and this can also be seen in the filtration results of this study. From Fig. 6 it can be seen that the contact angle decreased upon the addition of CNTs to the PES matrix, this was expected due to the hydrophilic enhancement by the –COOH and OH– groups as a result of acid functionalisation which improve water cluster formations^14^. It can be seen that the contact angle increases with the increase in CNT loading, however it decreases from 5 wt% CNT loading upwards, this is particularly due to increased CNT agglomeration within the pores^32^. The high contact angles are a result of the van der Waals forces between the PES matrix and CNTs as a result of high density due to increased CNT loading and subsequent increase in casting gel viscosity leading to the formation of slightly hydrophobic and less porous membranes^32^.

### Membrane performance evaluation

#### Effect of CNT loading on membrane performance

The flux and rejection of a membrane is largely impacted by the pore morphology, hydrophilicity and transmembrane pressure. Filtration experiments were conducted for the PES, PES/2.5 wt% CNT, PES/5 wt%, PES/7.5 wt% CNT and PES/10 wt% CNT. Each of the aforementioned membranes were tested at transmembrane pressures of 100 kPa, 300 kPa, 500 kPa, 700 kPa, 900 kPa and 1100 kPa, and by so doing the effect of pressure and increasing CNT loading on flux and rejection efficiency of industrial wastewater containing Cu, Fe, Ni, Zn and Cl traces was observed.

**Figure 7:**
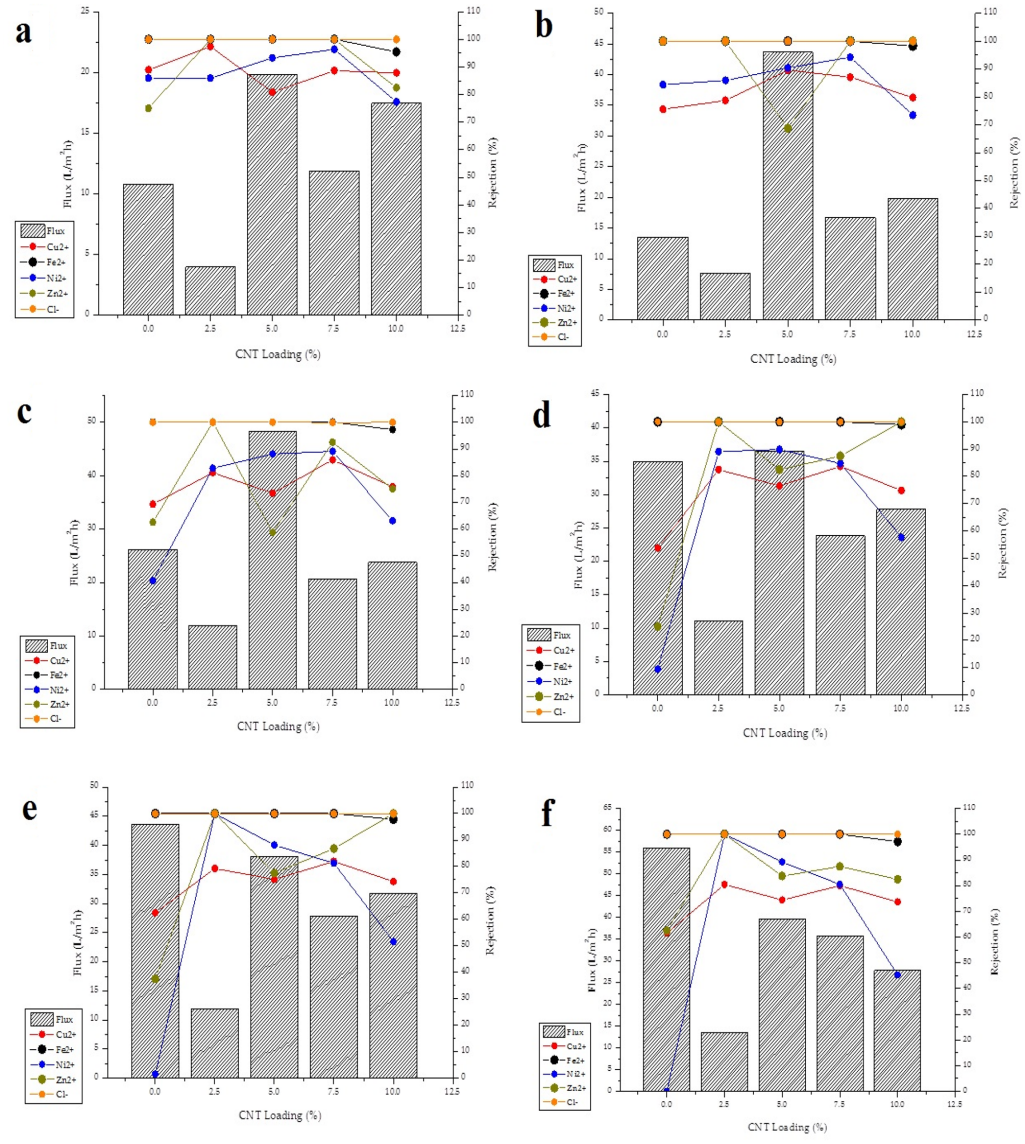
Membrane performance evaluation (**a**) evaluation at 100 kPa (**b**) evaluation at 300 kPa (**c**) evaluation at 500 kPa (**d**) evaluation at 700 kPa (**e**) evaluation at 900 kPa f) evaluation at 1100 kPa.

The performance evaluation by way of flux and rejection of all the membranes (pure PES, PES/2.5 wt% CNT, PES/5 wt%, PES/7.5 wt% CNT and PES/10 wt% CNT) is shown in Fig. 7(a), (b), (c), (d), (e), and (f) for respective transmembrane pressures of 100 kPa, 300 kPa, 500 kPa, 700 kPa, 900 kPa and 1100 kPa. It can be seen that for each membrane, the flux increases with increasing pressure irrespective of CNT loading. This was an expected result as the transmembrane pressure acts as the driving force which increases the capillary pressure thus pushing more volume of wastewater through the membrane over a short period of time^4,20,44^. The flux also significantly improves with increasing CNT loading largely owing to the hydrophilicity of the membrane due to the –COOH and OH– groups^14,15^. The PES membrane generated a flux of 10.8 L/m^2^h ± 8.79, 13.5 L/m^2^h ± 4.58, 26.2 L/m^2^h ± 5.01, 34.9 L/m^2^h ± 15.74, 43.7 L/m^2^h ± and 55.9 L/m^2^h ± 7.39 at 100 kPa, 300 kPa, 500 kPa, 700 kPa, 900 kPa and 1100 kPa respectively. The PES/5 wt% CNT and the PES/7.5 wt% CNT membranes had the most improved flux among the PES/CNT composites relative to the PES membrane. At pressures of 100 kPa, 300 kPa, 500 kPa, 700 kPa, 900 kPa and 1100 kPa, the PES/5 wt% CNT generated a flux of 19.8 L/m^2^h ± 13.47, 43.7 L/m^2^h ± 8.41, 48.3 ± 8.91 L/m^2^h, 36.5 L/m^2^h ± 3.37, 38.1 L/m^2^h ± 5.09 and 39.7 L/m^2^h ± 4.54; while the PES/7.5 wt% CNT membrane produced a flux of 11.9 L/m^2^h ± 11.14, 16.7 L/m^2^h ± 5.08, 20.6 L/m^2^h ± 15.89, 23.8 L/m^2^h ± 20.11, 27.8 L/m^2^h ± 7.77 and 37.7 ± 6.38 L/m^2^h.

At all the pressures (100 kPa, 300 kPa, 500 kPa, 700 kPa, 900 kPa and 1100 kPa), when CNTs are added to PES, the flux first decreases at 2wt% CNT loading, then increases until a maximum flux is reached at 5wt% CNT loading, thereafter a small decrease occurs at 7.5wt% and 10wt% CNT loading. This behaviour is consistent with the observation in the study of the membrane morphology in ”Section Effect of CNT on membrane morphology” where it was reported that the membrane pore size for the PES/2.5 wt% CNT, PES/5 wt% CNT, PES/7.5 wt% CNT and PES/10 wt% CNT membranes increased with 59%, 80%, 116% and 64% respectively, as a result of the formation of lesser closed cells in the asymmetric structure with larger macro voids^21^. The formation of larger pores as a result of accelerated exchange between the DMSO solvent and DI water during phase inversion consequently leads to a higher permeate flux^19,21,32^. The flux also improves because of the nano-channels formed between the CNTs and the PES matrix as a result of the lack of interaction between the CNTs and PES matrix due to low hydrophilic improvement^21^. However increased CNT loading coupled with high contact angles are a result of the van der Waals forces between the PES matrix and CNTs as a result of high density due to increased CNT loading and subsequent increase in casting gel viscosity and decreases the polymer free volume leading to the formation of slightly hydrophobic and less porous membranes with suppressed macro voids thus yielding a reduced flux^19,21,32^.

The rejection of can be seen to be the highest at a transmembrane pressure of 100 kPa, this is due to increased contact time between the PES membrane and the metal and salt ions such that there is enough adsorption time, this rejection capability decreases with increasing pressure due to the increased driving force^44^. This was an expected outcome because the increase in transmembrane pressure consequently increases the capillary pressure which acts the driving force for water transport through the membrane as it is pressure differential across the PES/CNT membrane—water interface^20^. The rejection for the PES membrane occurs by means of adsorption and by the Molecular Sieving Effect which works by taking into account the increased molecular sizes of Cu^2+^, Fe^2+^, Ni^2+^, Zn^2+^ and Cl^−^ in solution^45,46^. In the case of the PES/CNT composites, this increased contact time is particularly between the functionalised CNT and the metal and salt ions in the case of the PES/CNT composites. As a result of the functionalisation, the PES/CNT membranes gain a net negative charge as the wastewater had a pH of 9.5 and it is generally considered that the membrane charge becomes negative when the wastewater pH is higher than the iso-electric point (pH whereby the charge is neutral), and therefore by the Donnan Exclusion mechanism, they attract and adsorb Cu^2+^, Fe^2+^, Ni^2+^, Zn^2+^ and repel Cl^−^^15,18^. In fact, there is also a competition for places by the cations to attach on the membrane surface, buoyed by the influence of initial concentration of ions and number of contaminants present, therefore 3 mechanisms are at play, the molecular sieving effect, donnan exclusion mechanism and hydrophobic force^19^. There is a trade-off between flux and rejection in order to find the right balance as the 2 properties at the right operational pressure are inversely proportional, and as such it can be seen the flux decreases with increasing CNT loading as a result of the reduction in polymer free volume caused by the possible CNT agglomeration that comes with increasing CNT loadings^18,19,25,47,48^. The PES/5 wt% CNT membrane was the best performing membrane with an excellent flux-rejection trade-off balance at 300 kPa. The membrane generated a flux 43.7 L/m^2^h ± 8.41 whilst rejecting 89.6% ± 5.85 Cu, 100% ± 8.41 Fe, 90.5% ± 12.05 Ni, 68.8% ± 3.22 Zn and 99.99% ± 4.63 Cl.

## Conclusions

Functionalised CNT were incorporated into PES to successfully synthesize PES/2.5 wt% CNT, PES/5 wt% CNT, PES/7.5 wt% CNT and PES/10 wt% CNT membranes by phase inversion method. Pure PES membrane was also produced for comparison purposes. TEM was used to study the morphology of the CNT and it was confirmed that the CNT are MWCNT. XRD was used to study the effectiveness of the acid functionalisation, and to confirm that carboxylic groups were formed on the surface of the CNT. SEM morphology studies confirmed that the synthesised membranes are porous and that the pore size increases with increasing CNT loading. TGA studies indicated that the thermal stability increases with increasing CNT loading by virtue of the degradation temperature increasing, therefore the order of thermal stability was such that PES/7.5 wt% CNT > PES/5 wt% CNT > PES/2.5 wt% CNT > PES > PES/10 wt% CNT. Contact angle studies revealed that the hydrophilicity increased with increasing CNT loading as result of the successful acid functionalisation such the order of hydrophilicity was PES/2.5 wt% CNT > PES/7.5 wt% CNT > PES/10 wt% CNT > PES/5 wt% CNT > PES as result of CNT of the van der Waals forces between the PES matrix and CNTs as a result of high density due to increased CNT loading and subsequent increase in casting gel viscosity leading to the formation of slightly hydrophobic and less porous membranes.

Filtration tests were carried out by dead-end filtration cell for the PES, PES/2.5 wt% CNT, PES/5 wt% CNT, PES/7.5 wt% CNT and PES/10 wt% CNT membranes at 100 kPa, 300 kPa, 500 kPa, 700 kPa, 900 kPa and 1100 kPa. The PES/5 wt% CNT membrane was found to have the most improved results while having a good trade-off between flux and rejection at 300 kPa, generation a flux of 43.7 L/m^2^h whilst rejecting 89.6% Cu, 100% Fe, 90.5% Ni, 68.8% Zn and 99.99% Cl.

## References

[1] Atieh MA (2011). Removal of chromium (vi) from polluted water using carbon nanotubes supported with activated carbon. Proc. Environ. Sci..

[2] Das, R. Carbon nanotubes in water treatment. In *Nanohybrid catalyst based on carbon nanotube—A step-by-set guideline from preparation to demonstration*. Springer. Carbon Nanostructures. ISBN: 978-3-319-58151-4. 10.1007/978-3-319-58151-4_2 (2017).

[3] Das R (2014). Carbon nanotube membranes for water purification: A bright future in water desalination. Desalination.

[4] Maphutha S, Moothi K, Meyyappan M, Iyuke SE (2013). A carbon nanotube infused polysulfone membrane with polyvinyl alcohol for treating oil-containing wastewater. Sci. Rep.

[5] Ali S, Rehman SAU, Luan HY, Farid MU, Huang H (2019). Challenges and opportunities in functional carbon nanotubes for membrane-based water treatment and desalination. Sci. Total. Environ..

[6] Pourjafar S, Rahimpour A, Jahanshahi M (2012). Synthesis and characterization of pva/pes thin film composite nanofiltration membrane modified with TiO_2_ nanoparticles for better performance and surface properties. J. Ind. Eng. Chem..

[7] Jhaveri JH, Murthy ZVP (2016). A comprehensive review on anti-fouling nanocomposite membranes for pressure driven membrane processes. Desalination.

[8] Manawi Y (2016). Can carbon-based nanomaterials revolutionize membrane fabrication for water treatment and desalination. Desalination.

[9] Habiba U, Afifi AM, Salleh A, Ang BC (2017). Chitosan/(polyvinyl alcohol)/zeolite electrospun composite nanofibrous membrane for adsorption of Cr^6+^, Fe^3+^ and Ni^2+^. J. Hazard. Mater..

[10] Das R (2014). Multifunctional carbon nanotubes in water treatment: The present, past and future. Desalination.

[11] Zahid M, Rashid A, Akram S, Rehan ZA, Razzaq W (2018). A comprehensive review on polymeric nano-composite membranes for water treatment. J. Membr. Sci. Technol..

[12] Machodi MJ, Daramola MO (2019). Synthesis and performance evaluation of pes/chitosan membranes coated with polyamide for acid mine drainage treatment. Sci. Rep..

[13] Mathaba M, Daramola MO (2020). Effect of chitosan degree of deacetylation on the performance of pes membrane infused with chitosan during AMD treatment. Membranes.

[14] Celik E, Park H, Choia H, Choib H (2011). Carbon nanotube blended polyethersulfone membranes for fouling control in water treatment. Water. Res..

[15] Son M (2015). Efficacy of carbon nanotube positioning in the polyethersulfone support layer on the performance of thin film composite membrane for desalination. Chem. Eng. J..

[16] Wiley, D., & Weihs, G.F. Surface Charge Density. In Drioli, E., Giorno, L. (Eds.), *Encyclopedia of Membranes* (2016). Available from 31 August 2016. Accessed April 9th 2020. 10.1007/978-3-662-44324-8_2081.

[17] Wang Y (2016). Multi-walled carbon nanotubes with selected properties for dynamic filtration of pharmaceuticals and personal care products. Water Res..

[18] Ghiasi S, Behboudi A, Mohammadi T, Khanlari S (2019). Effect of surface charge and roughness on ultrafiltration membranes performance and polyelectrolyte nanofiltration layer assembly. Colloids. Surf. A: Physiochem. Eng. Asp..

[19] Vatanpour V, Madaeni SS, Moradian R, Zinadinim S, Astinchap B (2011). Fabrication and characterization of novel antifouling nanofiltration membrane prepared from oxidized multiwalled carbon nanotube/polyethersulfone nanocomposite. J. Membr. Sci..

[20] Rameetse MS, Aberefu O, Daramola MO (2020). Effect of loading and functionalization of carbon nanotubes on the performance of blended polysulfone/polyethersulfone membrane during treatment of wastewater containing phenol and benzene. Membranes.

[21] Wang L (2015). Fabrication and characterization of polyethersulfone/carbon nanotubes (pes/cnts) based mixed matrix membranes (mmms) for nanofiltration application. Appl. Surf. Sci..

[22] Nikita KM (2019). Understanding the morphology of MWCNT/PES mixed-matrix membranes using SANS: Interpretation and rejection performance. Appl. Water Sci..

[23] Chen, L., Xie, H., & Yu, W. Functionalization Methods of Carbon Nanotubes and Its Applications. In: Marulanda (Ed.), *Carbon Nanotubes Applications on Electron Devices*. **9**, 213–233 (2011). ISBN: 978-953-307-496-2. Available from:http://www.intechopen.com/books/carbon-nanotubes-applicationson-electron-devices/functionalization-methods-of-carbon-nanotubes-and-its-applications. Accessed September 28th 2020.

[24] Le VT (2013). Surface modification and functionalization of carbon nanotubes with some organic compounds. Adv. Nat. Sci. Nanosci. Nanotechnol..

[25] Shirazi Y, Tofighy MA, Mohammadi T (2011). Synthesis and characterization of carbon nanotubes/polyvinyl alcohol nano composite membranes for dehydration of isopropanol. J. Membr. Sci..

[26] Xu L, He J, Yu Y, Chen JP (2017). Effect of cnt content on physicochemical properties and performance of cnt composite polysulfone membranes. Chem. Eng. Res. Des..

[27] Mazov I, Kuznetsov VL, Simonova IA, Stadnichenko AI, Ishchenko AV, Romanenko AI, Tkachev EN, Anikeeva OB (2012). Oxidative behavior of multilayer carbon nanotubes of various diameters and morphology. Appl. Surf. Sci..

[28] Khani H, Moradi O (2013). Influence of surface oxidation on the morphological and crystallographic structure of multi-walled carbon nanotubes via different oxidants. J. Nanostruct. Chem..

[29] Das R, Hamid SBA, Ali MdE, Ramakrishna S, Yongzhi W (2015). Carbon nanotubes characterization by x-ray powder diffraction—A review. Curr. Nanosci..

[30] Salam MA, Burk R (2017). Synthesis and characterization of multi-walled carbon nanotubes modified with octadecyl amine and polyethylene glycol. Arab. J. Chem..

[31] Rojas JV, Toro-Gonzalez M, Molina-Higgins MC, Castano CE (2017). Facile radiolytic synthesis of ruthenium nanoparticles on graphene oxide and carbon nanotubes. Mater. Sci. Eng. B: Solid-state Mater. Adv. Technol..

[32] Phao N, Nxumalo EN, Mamba BB, Mhlanga SD (2013). A nitrogen-doped carbon nanotube enhanced polyethersulfone membrane system for water treatment. Phys. Chem. Earth..

[33] Manawi YM (2018). Engineering the surface and mechanical properties of water desalination membranes using ultra-long carbon nanotubes. Membranes.

[34] Zidan HM, Abdelrazek EM, Abdelghany AM, Tarabiah AE (2019). Characterization and Some Physical Studies of PVA/PVP filled with MWCNTs. J. Mater. Res. Technol..

[35] Zhao W, Li Y, Li Q, Wang Y, Wang G (2019). Investigation of the structure-property effect of phosphorus containing polysufone on decomposition and flame retardant epoxy resin composite. Polymers.

[36] Perng LH (2000). Thermal degradation mechanism of poly(arylene sulfone)s by stepwise py-gc/ms. J. Polym. Sci. A Polym. Chem..

[37] Khorshidi B, Hosseini SA, Ma G, McGregor M, Sadrzadeh M (2019). Novel nanocomposite polyethersulfone-antimony tin oxide membrane with enhanced thermal, electrical and anti-fouling properties. Polymer.

[38] Dhand V (2019). Fabrication of robust, ultra-thin and lightweight, hydrophilic pvdf-cnt membrane composite for salt rejection. Compos. B..

[39] Yudianti R, Onggo H, Saito Y, Iwata T, Azuma JI (2011). Analysis of functional group sited on multi-wall carbon nanotube surface. Open Mater. Sci. J..

[40] Corcione CE, Frigione M (2012). Characterization of nanocomposites by thermal analysis. Materials.

[41] Song P, Yu Y, Wu Q, Fu S (2012). Facile fabrication of hdpe-y-ma/nanodiamond nanocomposites via one-step reactive blending. Nanoscale. Res. Lett..

[42] Banerjee A, Ray SM (2020). Synthesis of novel composite membranes by in-situ intercalative emulsion polymerization for separation of aromatic-aliphatic mixtures by pervaporation. J. Membr. Sci..

[43] Huxtable ST (2003). Interfacial heat flow in carbon nanotube suspensions. Nat. Mater..

[44] Kaminska G (2015). Fabrication and characterization of polyethersulfone nanocomposite membranes for the removal of endocrine disrupting micropollutants from wastewater: Mechanisms and performance. J. Membr. Sci..

[45] Schaep J, Van der Bruggen B, Vandecasteele C, Wilms D (1998). Influence of ion size and discharge in nanofiltration. Sep. Purif. Technol..

[46] Chandrashekhar Nayal M (2020). Polyphenylsulfone/multiwalled carbon nanotubes mixed ultrafiltration membranes: Fabrication, characterization and removal of heavy metals Pb^2+^, Hg^2+^, and Cd^2+^ from aqueous solutions. Arab. J. Chem..

[47] Domenech B (2012). Bifunctional polymer-metal nanocomposite ion exchange materials. Ion. Exch. Technol..

[48] Gumbi NN, Li J, Mamba BB, Nxumalo EN (2020). Relating the performance of sulfonated thin-film composite nanofiltration membranes to structural properties of macrovoid-free polyethersulfone/sulfonated polysulfone/o-mwcnt supports. Desalination.

